# Early Warning of Abnormal Operating Modes via Feature Extraction from Cross-Section Frame at Discharge End for Sintering Process

**DOI:** 10.3390/s25144267

**Published:** 2025-07-09

**Authors:** Xinzhe Hao, Sheng Du, Xian Ma, Mengxin Zhao

**Affiliations:** 1 School of Automation, China University of Geosciences, Wuhan 430074, China; xinzhe_hao@163.com (X.H.); xianma@cug.edu.cn (X.M.); 15505345126@163.com (M.Z.); 2 Hubei Key Laboratory of Advanced Control and Intelligent Automation for Complex Systems, Wuhan 430074, China; 3 Engineering Research Center of Intelligent Technology for Geo-Exploration, Ministry of Education, Wuhan 430074, China

**Keywords:** abnormal operating mode, feature extraction, early warning model, sintering process

## Abstract

Abnormal operating modes in the iron ore sintering process often lead to reduced productivity and inferior sinter quality. The timely early warning of such modes is therefore essential in maintaining stable production and ensuring product quality. To this end, we develop an early warning approach that integrates cross-sectional image features from the discharge end. First, an edge detection-based scheme is designed to isolate and analyze the red fire layer in the image. Second, a random forest feature importance ranking is employed to select process variables. Third, a Bayesian neural network is trained on both selected process variables and visual features extracted from the red fire layer to construct the early warning model. Finally, the burn-through point is adopted as the classification criterion, and experiments are carried out on raw data collected from an industrial plant. The results demonstrate that the proposed method enables the accurate early detection of abnormal operating modes, achieving accuracy of 94.07%, and thus holds strong potential for industrial application.

## 1. Introduction

Sintering is the principal method of producing high-quality synthetic iron ore. During sintering, the base ore is blended with other materials to enrich desirable components and, through high-temperature reactions, to reduce impurities, thereby upgrading the product quality. The sinter ore produced through this process serves as the primary burden material for blast furnace ironmaking [1]. The global crude steel output reached 1.8826 billion tons in 2024 [2]. As the steel demand continues to rise, high-grade natural ore alone can no longer satisfy industrial needs, making the sintering process increasingly critical.

Sintering is a thermal agglomeration operation whose principal feed materials are iron ore, returning sinter ore, fluxes and coke [3]. Its high energy consumption and severe environmental impact have long been major concerns for steel plants. An operating mode describes the state of process variables under specific conditions. Operators adjust control actions according to different modes to improve product quality, boost productivity and reduce energy use. Correctly identifying operating modes in the sintering process therefore has substantial economic value.

This study aims to address the following technical question: can the combustion state of a sintering strand—specifically, deviations from normal operating modes—be accurately predicted one minute in advance based on image and process data? To this end, we propose a model combining visual feature extraction with probabilistic learning to provide early warnings of over-burning or under-burning.

The recognition of abnormal operating modes has attracted considerable research attention, enabling mode recognition methods to be applied to many industrial processes, including the sintering process [4]. Since manual monitoring is inherently limited in frequency, scope and accuracy [5], it may fail to promptly detect process deviations, thereby compromising production safety. Hence, recognizing abnormal operating modes in industrial processes is crucial for operational safety. Tang et al. proposed an attention-based early warning framework for abnormal operating conditions in FCCU, combining denoising, Conv-LSTM and anomaly attention modules to improve the detection accuracy and robustness [6]. Du et al. clustered and smoothed sintering data with fuzzy C-means, built Naïve Bayes sub-models for mode recognition and combined them into a high-accuracy model, laying the groundwork for stable sinter quality [7]. However, their work did not consider the cross-section frame at the discharge end.

Recent studies have placed considerable emphasis on analyzing discharge end images in the context of iron ore sintering. In [8], a hybrid just-in-time soft sensing system was introduced to estimate the carbon efficiency by extracting key features from cross-sectional frames. A genetic algorithm-based fuzzy c-means clustering technique was applied to segment key frames into distinct regions, which then served as inputs for a soft sensor to predict the comprehensive carbon ratio (CCR) in real time. Liang et al. [9] proposed a CNN–Transformer dual-stream network for the classification of sinter flame combustion states with high accuracy. However, these approaches primarily address either combustion characteristics or efficiency analysis, rather than the early warning of abnormal operating modes.

Although the above methods have achieved good results in combustion or efficiency analysis, they do not utilize discharge end images to recognize or issue early warnings of abnormal operating modes. In this study, early warning refers to the prediction of abnormal operating modes before they fully emerge, enabling operators to intervene proactively and reduce the duration of unstable states on the strand. Moreover, ignoring image features tends to reduce the accuracy in recognizing abnormal operating modes, as these features provide direct visual cues about combustion states at the discharge end.

To address the challenges in the early detection of abnormal operating modes, this study proposes an early warning model based on feature extraction from cross-sectional frames—infrared snapshots captured at the sinter cake’s discharge end that reveal a vertical section of the combustion zone. The approach begins with frame segmentation to isolate the red fire layer, which is then analyzed separately. The Sobel edge detection algorithm is employed due to its balance of speed and accuracy, making it suitable for real-time industrial applications [10]. Subsequently, a one-way analysis of variance reveals that the continuity and height of the red fire layer are highly correlated with the operating mode. To further refine input selection, random forest feature importance analysis is performed, identifying the most relevant process variables. These top-ranked variables are used as inputs to a Bayesian neural network (BNN) model, which is trained to issue early warnings of abnormal operating modes. Real production data from an industrial sintering plant are utilized for model training and validation. Experimental results demonstrate that the proposed model achieves prediction accuracy exceeding 94.07%, significantly outperforming the existing methods referenced in [7,11].

The key contributions of this paper are summarized as follows:

(1) An early warning method for abnormal operating modes that exploits feature extraction from the cross-section frame at the discharge end;
(2) A labeled, interpretable early warning model that operators can readily accept for control guidance;
(3) An early warning approach that combines Bayesian theory with operator experience, improving the reliability of abnormal mode forecasting.

## 2. Process Description and Design Method

This section first gives a detailed account of iron ore sinter production and of the characteristics of the cross-section frame at the discharge end. On the basis of the production requirements, an early warning scheme for abnormal operating modes is then formulated.

### 2.1. Description of the Iron Ore Sintering Process

**Figure 1:**
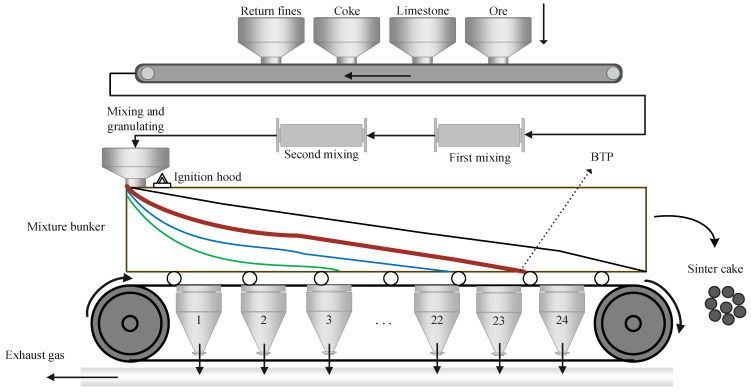
Iron ore sintering process. “BTP” denotes the burn-through point, where the combustion zone reaches the bottom of the sinter bed. Curved colored lines represent the movement of the sinter cake along the strand, with different colors distinguishing various process segments for clarity.

The iron ore sintering process is multivariate, highly nonlinear and strongly time-delayed, and it can switch among several operating modes [12]. The Dwight–Lloyd sintering machine remains the dominant equipment used in current sintering operations. In this study, a 360 m^2^ strand with 24 bellows is selected as the case example. The overall iron ore sintering process is illustrated in Figure 1.

The process agglomerates iron ore, returning sinter ore, fluxes and coke into the hot sinter ore [13]. The raw materials are first blended with water in fixed proportions to form a mixture that is stored in a mixing hopper. A roll feeder then distributes the mixture onto a moving pallet strand. Inside the ignition hood, the charge is ignited, after which down-draft fans draw combustion air so that the bed burns progressively from top to bottom. When the leading edge of the combustion zone reaches the grate bars, the mixture has burned out and the process reaches the burn-through point (BTP); combustion then ceases. Under the normal operating mode, the BTP coincides with approximately the 23rd bellows.

Operators judge process stability mainly by tracking the BTP. Because the BTP marks the location where the mixture has finished burning, its position is the most important thermal indicator. If the BTP appears upstream of the target position, the effective bed area is not fully utilized and strand productivity falls. If the BTP appears downstream, the bed has not burned through at discharge, which increases the recycle load of returning the sinter ore. Hence, the current operating mode can be directly assessed from the BTP position [11].

**Figure 2:**
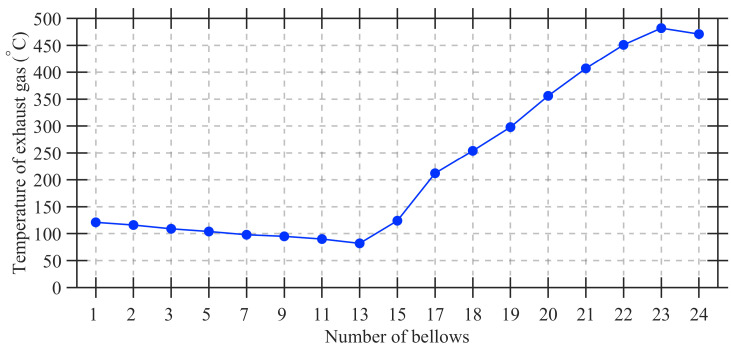
Temperature of the exhaust gas in the bellows.

After complete combustion, the hot bed travels roughly one additional bellows length and reaches the segmentation frame. Here, the porous sinter cake is broken loose and discharged. When this solid mass has completely fallen away, the cross-section frame at the discharge end becomes fully visible, providing the image used in this study. A typical bellows exhaust gas temperature profile is shown in Figure 2.

### 2.2. Characteristic Analysis of Sintering Process

Before applying the cross-section frame at the discharge end for the early warning of abnormal operating modes, it is essential to examine its correlation with the operating mode and to clarify the challenges involved in accurate early warning.

At the discharge end, the captured cross-section frame reveals three distinct zones: the red fire layer, the background area and the trolley baffle. Thermally, the strand can run in one of three operating modes—over-burning, normal or under-burning [14]. Over-burning arises when the cake reaches burn-through ahead of schedule, pushing the BTP upstream. Under-burning occurs when combustion remains incomplete, so the BTP shifts downstream. Either deviation diminishes strand utilization and lowers the sinter quality, thereby impairing subsequent blast furnace performance [15].

The red fire layer offers a clear visual indicator of the combustion state. Its height and continuity are key descriptors. A tall, unbroken red band suggests an elevated combustion front and under-burning; a shallow, intermittent band indicates premature burn-through and over-burning. In normal operation, both attributes appear moderate, reflecting a combustion zone that terminates at the appropriate depth. Each operating mode, therefore, corresponds to a distinct red fire layer pattern.

### 2.3. Burn-Through Point as the Classification Criterion

The location of the BTP ($L_{BTP}$) is taken as the indicator of the operating mode. In normal operation, $L_{BTP}$ must lie inside the band ${[L}_{d}-d,L_{d}+d]$, where $L_{d}$ is the target BTP position and d the admissible fluctuation (here, d=0.5 and $L_{d}=22.5$). If $L_{BTP}$ rises above the upper limit, the bed is underburned; if it falls below the lower limit, the bed is over-burned.

Thus, three modes are defined:

$${OM}_{1}\text{—}\mathrm{Over-burning:}\;L_{BTP}<L_{d}-d.$$

Premature burn-through lowers strand utilization and reduces the sinter output:

$${OM}_{2}\text{—}\mathrm{Normal:}\;L_{d}-d\leq L_{BTP}\leq L_{d}+d.$$

The mixture burns out precisely at the desired location, fulfilling the production requirements:

$${OM}_{3}\text{—}\mathrm{Under-burning:}\;L_{BTP}>L_{d}-d.$$

This condition negatively affects both the output and sinter quality.

In over-burning conditions, combustion finishes prematurely near the grate, resulting in a low and fragmented red fire layer. In contrast, under-burning keeps the combustion zone in the upper–middle bed, where the red fire layer appears tall and continuous. During normal operation, the layer is located in the middle–lower section of the frame with stable continuity.

### 2.4. Design of the Early Warning Scheme for Abnormal Operating Modes

Given the strong nonlinearity and high degree of coupling inherent in the sintering process [16], this study proposes an early warning model for abnormal operating modes that leverages the cross-section frame at the discharge end. To ensure reliable early warning, it is therefore crucial to fully incorporate the relevant state parameters into the model. In particular, because the key frame features are also highly correlated with the operating mode, they must be included among the model inputs.

**Figure 3:**
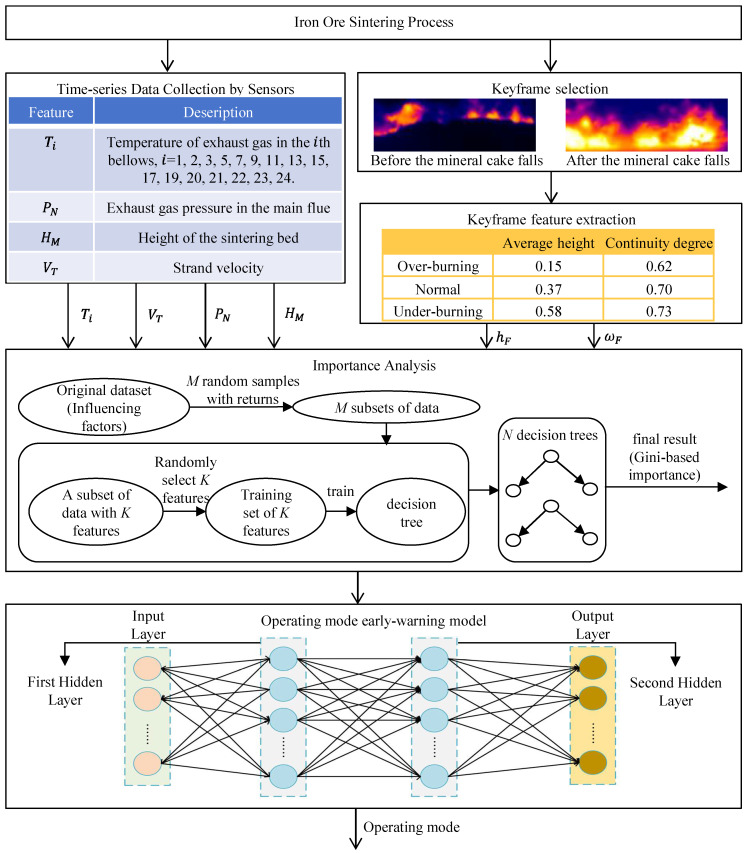
Framework of the early warning system for the detection of abnormal operating modes in the sintering process.

The early warning scheme comprises three main steps. First, an image processing module is designed to extract feature parameters from the discharge end cross-section frame. Next, a random forest feature importance ranking is applied to select the variables. Subsequently, a BNN is constructed to serve as the early warning model. The overall structure of the proposed scheme is illustrated in Figure 3 for better clarity.

Furthermore, for the model inputs, the key frame features are extracted first. In view of the periodic descent of the sinter cake, a key frame selection strategy is devised. Once the discharge end key frame is selected, the Sobel operator is subsequently applied to segment it into the red fire layer and the background region. The resulting feature parameters are calculated and stored in a database.

To handle the multifactor influences on the operating mode, a random forest algorithm is employed to compute the importance of each variable. Moreover, random forest is simple, easy to implement and incurs only a modest computational cost; variables with higher importance scores are retained as inputs to the early warning model.

Finally, based on the selected features, a BNN-based early warning model is developed, which takes as inputs the feature parameters of the discharge end frame together with several key state parameters of the sintering process. The BNN is chosen because it merges the strengths of Bayesian statistics and neural networks, thereby enhancing both the forecast accuracy and generalization ability.

## 3. Early Warning Model for Abnormal Operating Modes

This section establishes the early warning model for abnormal operating modes. First, an image processing module is used to extract feature parameters from the cross-section frame at the discharge end. Second, a random forest feature importance ranking is applied to select the input variables. Finally, a BNN is trained to construct the early warning model.

### 3.1. Feature Extraction from the Cross-Section Frame at the Discharge End

We develop a key frame selection method based on the characteristics of the infrared video captured at the discharge end. The key frame extraction procedure processes the images captured at the discharge end in order to identify those moments that best represent changes in the operating mode. A camera mounted at the discharge end continuously records the sintering process, and, during processing, only the key frames that have a significant influence on the operating mode are retained.

**Figure 4:**
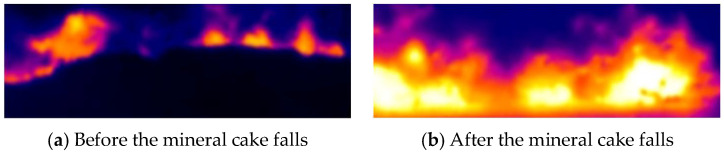
Comparison of sintered ore cake before and after falling.

The sinter cake falls from the pallet at roughly 60 s intervals; immediately afterwards, a large dust plume is generated, degrading the image quality. To avoid this problem, the key frame is extracted during the initial appearance of the red fire layer, before dust obscures the view. In the present study, an image is captured every 5 s, and the gray-level difference between successive frames is calculated. The frame with the maximum difference is selected as the key frame because it clearly reflects the operating mode while minimizing dust interference, ensuring both efficiency and accuracy. A comparison of the frame before and after the sinter cake has fallen is shown in Figure 4.

Information from the red fire layer is essential for early warning [17], so this region must be segmented before the image features are calculated. Sobel edge detection, a classical image processing algorithm, identifies edges by evaluating the gradient magnitude and direction at each pixel, and it is applied in this study to segment the key frames.

To reduce noise before segmentation, each RGB frame is converted to grayscale and smoothed with a 3 × 3 Gaussian filter (σ=1.0). Then, Sobel edge detection is applied to compute image gradients in both the horizontal and vertical directions. Edge points are retained where the gradient magnitude exceeds 80, followed by non-maximum suppression to thin the edges.

Using the resulting edge map, the red flame region is segmented by identifying the upper boundary of the red zone. From this boundary, the average height h is calculated as the mean of the topmost red pixels in each column. The continuity degree *ω* is defined as the ratio of columns where the flame height is greater than or equal to h, capturing the horizontal consistency of the red region. These features quantitatively reflect the vertical size and spatial uniformity of the red flame region.

The average height is obtained by

(1) $$\begin{array}{c} h=\frac{1}{k}\sum_{i=1}^{k}y_{i} \end{array},$$

where $y_{i}$ denotes the highest pixel ordinate in the i-th column and k is the total number of columns.

Continuity is measured via

(2) $$\begin{array}{c} \omega =\frac{n_{p}}{k} \end{array},$$

with $n_{p}$ representing the count of columns whose red fire layer height reaches (or exceeds) that average value, while k again is the column total.

Thus, h captures the vertical extent of the red zone, whereas ω quantifies how consistently that height is maintained across the frame.

### 3.2. Input Variable Selection of Early Warning Model

The sintering dataset contains 20 measured variables, as shown in Figure 3. To address the multifactor influence on the operating mode, a random forest algorithm is employed to evaluate the importance of each variable. Random forest is an ensemble method that builds multiple decision trees as base learners and aggregates their outputs to improve the performance. It improves the classification performance of single trees by bootstrap aggregation and random feature splitting during tree construction [18]. It is straightforward to implement and has a low computational cost.

To quantify the contribution of each variable to the classification process, the Gini index is used as the splitting criterion in the random forest algorithm. For any node *m* in a decision tree within the random forest, the Gini impurity $g_{m}$ is defined as

(3) gm=∑k=1Kp^mk(1−p^mk),

where K is the total number of classes, and p^mk is the estimated probability that a sample at node *m* belongs to class *k*.

For binary classification problems, this can be simplified as

(4) gm=2p^m(1−p^m).

If a variable $t_{j}$ is used in the i-th decision tree and contributes to M splits, then the importance score $v_{ij}$ of variable *t_j_* in that tree, based on the reduction in Gini impurity, is given by

(5) $$v_{j}^{\left(Gini\right)}=\sum_{m=1}^{M}\Delta g_{jm}^{\left(Gini\right)}.$$

where $\Delta g_{jm}^{\left(Gini\right)}$ is the reduction in Gini impurity at node m when splitting on variable $t_{j}$.

The total importance of variable $t_{j}$ across all n trees in the random forest is calculated as the average:

(6) $$v_{j}^{\left(Gini\right)}=\frac{1}{n}\sum_{i=1}^{n}v_{ij}^{\left(Gini\right)}.$$

To implement feature selection, a random forest classifier was constructed using 100 decision trees, with each tree limited to a maximum depth of 10 to avoid overfitting and ensure interpretability. The Gini index was used as the splitting criterion at each node. After training, the Gini importance score of each variable was computed by aggregating its contribution to Gini impurity reduction across all trees in the ensemble, as described in Equations (3)–(6).

Variables whose importance scores exceeded a predefined threshold of 0.3 were selected as relevant inputs to the early warning model. These include the exhaust gas temperatures at the 23rd and 21st bellows ($T_{23}$, $T_{21}$), the strand speed, the main flue negative pressure, and the bed height. The final selection results and their respective importance values are listed in Table 1.

To accelerate the early warning model, the number of selected inputs must be limited. In order to avoid excessive redundancy while still retaining sufficient time series information, an importance score threshold of 0.3 is adopted; the resulting scores are listed in Table 2. The analysis shows that the temperature of the exhaust gas in the 23rd bellows ($T_{23}$), $T_{21}$, the strand speed, the main flue negative pressure, and the bed height all have importance values above the threshold. These five variables are therefore chosen as the inputs to the abnormal operating mode early warning model.

To further enhance the sensitivity and robustness of the early warning model, visual features extracted from the flame region are integrated with the selected process variables. Specifically, two visual descriptors—the average height and the continuity degree—are derived from key frames, as detailed in Section 3.1. These image-based indicators complement the physical sensor data by capturing the thermal and spatial characteristics of the sintering process, which are difficult to obtain through conventional measurements.

The fusion of visual and process data is achieved through minute-level temporal alignment. Since both the image frames and the process logs are timestamped, we convert their respective timestamps into a standardized datetime format and down-sample them to a minute resolution. Only the records with matched timestamps across both sources are retained. This ensures strict synchronization between visual indicators and process readings, allowing the model to learn from coherent, multimodal input.

The final input to the early warning model therefore consists of a 7-dimensional vector at each time step, including the five selected process variables ($T_{23}$, $T_{21}$, strand speed, main flue negative pressure, and bed height) and two visual features (average height and continuity degree). These features are directly concatenated without further normalization, as their value ranges are already comparable and stable across time. This unified representation enables the model to simultaneously learn from physical measurements and flame dynamics, thereby improving the early detection of abnormal operating modes.

### 3.3. Structure of Early Warning Model

The early warning model for sintering operating modes is designed to infer the upcoming state ${OM}_{k}$ ($k\in \left\{1,\;2,\;3\right\}$) using a set of real-time process parameters. The selected parameter vector $P_{\mathrm{in}}\in R^{d}$, obtained from prior feature selection, serves as the model input, and the forecast result represents the output.

To address the limitations of traditional modeling, BNNs integrate the interpretability of Bayesian statistics with the expressive power of deep learning [19]. While traditional Bayesian models rely on Bayes’ theorem to update probability estimates by combining prior distributions with observed data, they often struggle with scalability in high-dimensional, nonlinear contexts. Neural networks, on the other hand, are effective in abstracting complex patterns but lack inherent uncertainty quantification. BNNs overcome both limitations by treating all weights and biases as probability distributions rather than fixed values, allowing them to represent model uncertainty and reduce overfitting.

Formally, the transformation in each layer l of a BNN is defined as

(7) $$a^{\left(l\right)}=\sigma \left(W^{\left(l\right)}a^{\left(l-1\right)}+b^{\left(l\right)}\right),$$

where $W^{\left(l\right)}$ and $b^{\left(l\right)}$ are random variables drawn from prior distributions, σ(⋅) denotes the activation function and $a^{\left(0\right)}=P_{\mathrm{in}}$ is the input feature vector.

To approximate the intractable posterior p(W,b|D), variational inference is used:

(8) $$p\left(W,b|D\right)\approx q\left(W,b|\theta \right),$$

where q(⋅) is a tractable variational distribution parameterized by θ, and D is the dataset.

The objective is to minimize the evidence lower bound (ELBO):

(9) $$L=E_{q}\left[\log p\left(D|W,b\right)\right]-KL\left(q\left(W,b\right)∥p\left(W,b\right)\right),$$

**Figure 5:**
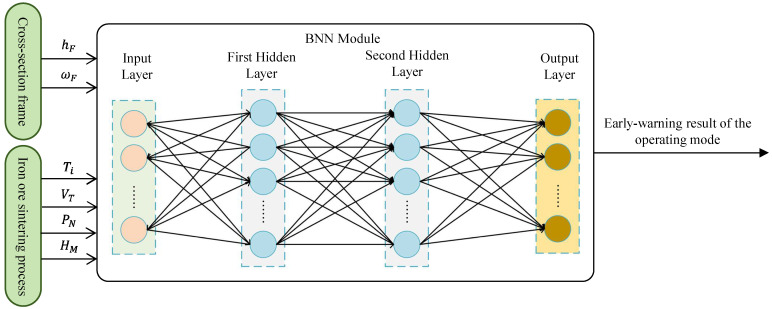
Overall architecture of the proposed model for the early detection of abnormal operating modes in the sintering process.

This balances data fit and model complexity, enabling better generalization and robustness. The architecture of the complete early warning system is illustrated in Figure 5.

To implement the early warning model, we constructed a BNN composed of three fully connected hidden layers with 64, 32 and 16 neurons, respectively. All hidden layers use the ReLU activation function. To prevent overfitting, a dropout layer with a rate of 0.2 is applied after each hidden layer.

The model is trained using the Adam optimizer with a learning rate of 0.001 for 200 epochs. Variational inference is applied to approximate the intractable posterior distributions over weights and biases. To ensure generalization, a 5-fold cross-validation strategy is used during training and model selection. This configuration provides a balance between expressiveness, uncertainty estimation and computational efficiency.

## 4. Experimental Study and Analysis

This section validates the proposed alarm method for abnormal operating modes by means of experiments based on raw data collected from a steel plant and discusses the results.

### 4.1. Experimental Design

To validate the effectiveness of the proposed early warning scheme utilizing cross-section frame features at the discharge end, a series of images at the discharge end were collected from an actively operating industrial strand. Key frames were identified in the infrared video—captured with IRTool Pro—by applying the selection strategy described above. After saving the raw stream to a local directory, a Python routine (i) isolated the key frames, (ii) defined crop coordinates and (iii) automatically clipped the region of interest, namely the red fire layer. Each cropped frame was then passed through image segmentation and feature extraction steps, and the resulting parameters were written back to disk by the same Python script.

The dataset used in this study was collected from a large-scale sintering production line located in Central China, which processes magnetite-based mixed ores. The data spanned a continuous 14-day operating period in October 2023. Ground truth labels for each operating mode—under-burning, normal and over-burning—were manually annotated based on a synchronized analysis of the flame morphology (from infrared images), bed temperature profiles and exhaust gas composition.

Due to confidentiality agreements with the plant operator, the original data cannot be made publicly available. Nevertheless, the dataset is representative of real industrial scenarios and was annotated through cross-validation with multiple independent indicators, ensuring its credibility.

Because the field sensors sampled every 5 s, the sampling interval of the raw production data was enlarged to 1 min to reduce the influence of high-frequency noise. In total, 3540 samples were extracted. Of these, 3000 were used to construct a balanced training set (1000 per class), while 540 samples (180 per class) were held out for testing. During model development, 10% of the training set was used as a validation set for hyperparameter tuning. A stratified sampling strategy ensured that the training, validation and test subsets all preserved the class balance.

**Figure 6:**
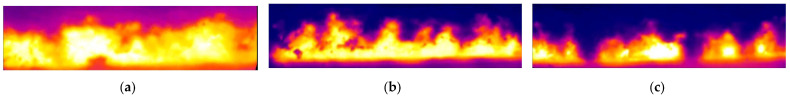
Representative cross-section frames at the discharge end under different sintering conditions. (**a**) original figure (under-burning); (**b**) original figure (normal); (**c**) original figure (over-burning).

We segmented each key frame using the Sobel edge detection method. Frames corresponding to the three operating modes—identified in consultation with experienced plant operators—were selected for segmentation. The results are presented in Figure 6, where it is evident that the red fire layer exhibits distinct characteristics across the three operating modes: under over-burning, the layer’s continuity and the average height are markedly lower than in the normal mode, whereas under-burning yields the greatest height and continuity. The numerical feature parameters for the three frames depicted in Figure 6 are provided in Table 2.

To refine the experimental results, multiple runs were performed. Once the features of the cross-section frame at the discharge end had been extracted, they were merged with the time series process data to serve as the input vector of the abnormal operating mode early warning model, which then produced the forecast result for the operating mode.

To assess the feasibility of real-time deployment, the computational performance of the proposed BNN model was evaluated. All experiments were conducted in a Python 3.10.0 environment under Windows 10 Professional, using a 12th Gen Intel i7-12700H processor and 16 GB RAM (Intel Corporation, Santa Clara, CA, USA), without GPU acceleration. The average inference time per sample was approximately 50 ms, including the variational forward pass. Given that the system operates on a one-minute sampling interval, this latency is negligible, confirming that the model can be deployed in real-time applications without requiring high-end hardware.

The forecast performance for different operating modes is represented by the confusion matrix in Table 3. In this matrix, $M_{ij}(i=1,2,3;j=1,2,3)$ denotes the number of test samples that are predicted as operating mode ${\mathrm{OM}}_{i}$ by the early warning model, while their true label is ${\mathrm{OM}}_{j}$. In addition, three metrics are defined to evaluate the forecast performance: $\eta_{a}$ represents the overall accuracy, $\eta_{f}$ the false alarm rate and $\eta_{m}$ the missing alarm rate. They are expressed as

(10) $$\eta_{a}=\frac{M_{11}+M_{22}+M_{33}}{\sum_{i=1}^{3}\sum_{i=1}^{3}M_{ij}}\times 100\%;$$

(11) $$\eta_{f}=\frac{M_{12}+M_{32}}{\sum_{i=1}^{3}M_{i2}}\times 100\%;$$

(12) $$\eta_{m}=\frac{M_{21}+M_{31}+M_{13}+M_{23}}{\sum_{i=1}^{3}M_{i1}+\sum_{i=1}^{3}M_{i3}}\times 100\%.$$

### 4.2. Experimental Result Analysis

The operating mode defined by $L_{BTP}$ is used as the recognition target. To evaluate the forecast accuracy more effectively, the metrics introduced above are applied to verify the validity of the proposed early warning model for abnormal operating modes.

To further evaluate the individual contributions of sensor data and image features, an ablation study was conducted. Three input configurations were tested: (1) sensor data only, (2) image features only and (3) the full model combining both. When only sensor data were used, the classification accuracy dropped to 88.6%, showing limited discriminative power. Using only image features achieved a moderately better result of 83.2%. In contrast, the proposed model integrating both modalities reached the highest accuracy of 94.07%. These results confirm that each modality contributes distinct information, and their fusion yields a significant performance gain. This validates the effectiveness of the feature extraction strategy from the discharge-end cross-section frame in complementing traditional process parameters for more accurate operating mode recognition.

**Figure 7:**
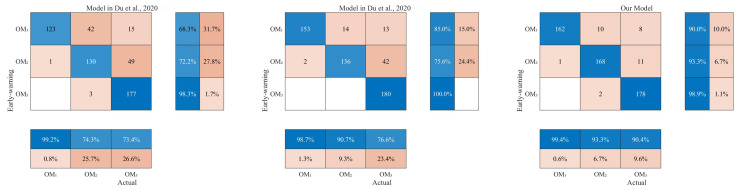
Confusion matrices of results for three early warning models [7,13].

In addition, we conducted two comparative experiments to highlight the advantages of the proposed method based on feature extraction from the cross-section frame at the discharge end. The model presented in [7] targets operating mode recognition in the iron ore sintering process and employs a Naïve Bayes classifier, making it comparable to our method in terms of its probabilistic framework. The model in [13] employs a fuzzy rule-based framework to predict operating modes for the same process, sharing essentially the same background and objective as the present study. Therefore, we adopt these two models as benchmarks for comparison with our proposed approach. According to Table 4 and Figure 7, our model exhibits the best performance in terms of the mean accuracy. To ensure practical value, a reliable early warning model should combine high accuracy with both a low false alarm rate and a low missing alarm rate. Our model surpasses the models in [7,13] in all three metrics—accuracy, the false alarm rate and the missing alarm rate. Hence, the proposed approach is effective. It offers operators a reliable reference for the adjustment of the strand operation and thus holds significant practical value in enhancing the combustion efficiency and boosting productivity in the sintering process.

**Figure 8:**
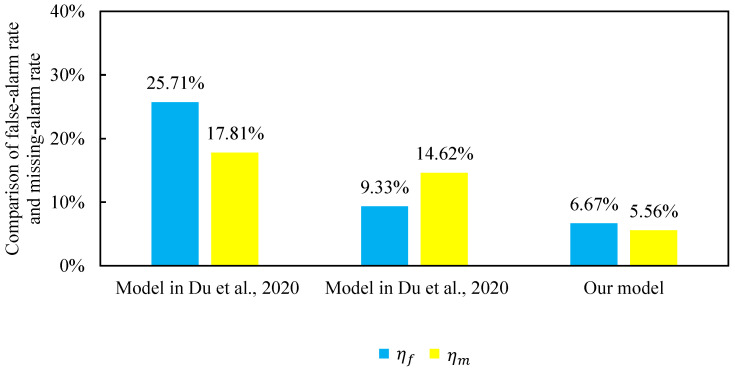
Comparison of false alarm rate and missing alarm rate among different algorithms [7,13].

A comparison of the false alarm rate and missing alarm rate results is shown in Figure 8. Our model achieves both a lower false alarm rate $\eta_{f}$ and missing alarm rate $\eta_{m}$ compared to the benchmarks. The false alarm rate increases the computational burden and may trigger unnecessary control actions, while the missing alarm rate risks failing to address actual abnormal conditions. Since intelligent decision-making depends on the early warning outputs, reducing $\eta_{m}$ is especially critical in maintaining process stability. The comparison between the false alarm and missing alarm rates across different algorithms is shown in Figure 8. It is evident that the proposed model outperforms the benchmark methods in both the false alarm and missing alarm rates.

It should be noted that, although the proposed system does not directly intervene in the process, it predicts abnormal conditions one minute in advance, offering timely guidance for operators to take preventive actions. This aligns with practical definitions of early warning in industrial process monitoring.

### 4.3. Significance Test

To evaluate whether the observed differences in recognition performance across the three models—the model in [7], the model in [13] and the proposed model—were statistically significant, a chi-squared ($\chi^{2}$) test was performed based on the numbers of valid and invalid predictions. The procedure is detailed below.

Step 1. Hypothesis setting

**H_0_:**  *There is no significant difference in recognition performance among the three models.*

**H_1_:**  *At least one model performs significantly differently.*

Step 2. Test statistic computation

The chi-squared test statistic is defined as

(13) $$\begin{array}{c} \chi^{2}=\sum \frac{(A_{rc}-T_{rc}{)}^{2}}{T_{rc}} \end{array}\;,$$

where $A_{rc}$ and $T_{rc}$ represent the actual and expected frequencies in the *r*-th row and *c*-th column, respectively. The expected frequency is computed by

(14) $$T_{rc}=\frac{N_{r}\cdot N_{c}}{N}\;,$$

with $N_{r}$ and $N_{c}$ denoting the row and column totals and N the overall sample size.

Based on the recognition results of 540 samples per model (1620 in total), the calculated test statistic is

(15) $$\chi^{2}=49.33,$$

Step 3. Significance level and decision

The degrees of freedom for a 3 × 2 contingency table are

(16) df=(3−1)×(2−1)=2,

and, at a significance level of α=0.01, the critical value from the chi-squared distribution table is

(17) $$\chi_{0.01}^{2}(2)=9.21.$$

Since

(18) $$\chi^{2}=49.33>9.21\Rightarrow p<0.0001,$$

the null hypothesis is rejected. Therefore, we conclude that there is a statistically significant difference in recognition performance among the three models. Specifically, the proposed model achieves the highest valid recognition rate and lowest error rate, indicating its superior classification effectiveness in the early warning task.

## 5. Conclusions

We emphasize that the reliable early warning of abnormal operating modes is vital in accurately characterizing the combustion state of a sintering strand and maintaining stable process control. In this study, we began by devising a dedicated feature extraction routine for the discharge end cross-section frame, and then we built an early warning model for abnormal operating modes based on a BNN. Our early warning model achieves higher accuracy than other benchmark models. Overall, the early warning model that relies on image features from the discharge end constitutes an effective guide for sinter production and offers tangible benefits for plants aiming to cut costs while boosting operational efficiency.

Differences in equipment design, sensor placement or operational control strategies may impact performance. However, the proposed method relies on high-level features such as the flame structure and core process indicators (e.g., strand speed, bed height), which are common in most sintering operations. We believe that the method can be adapted to other systems through moderate retraining. Future work will focus on cross-site generalization using domain adaptation or transfer learning techniques. Looking ahead, the framework could be coupled with an intelligent control layer to adjust the operating mode automatically.

Despite its high-precision warning performance, the approach still has some notable shortcomings:

(1) It relies on an on-site, high-temperature infrared camera to obtain the key frames required for real-time warnings;
(2) Because of the harsh working environment, discharge end images are sometimes blurred, which can degrade the accuracy of the warning results;
(3) While the model demonstrates high accuracy on a specific sintering strand, its direct applicability to other plants or configurations has not yet been validated.

## Figures and Tables

**Table 1:** Results of importance analysis.

**Feature**	$T_{23}$	$V_{T}$	$T_{21}$	$P_{N}$	$H_{M}$	$T_{22}$	$T_{24}$
Gini-based importance	0.402	0.356	0.341	0.305	0.302	0.282	0.279
**Feature**	$T_{17}$	$T_{20}$	$T_{11}$	$T_{1}$	$T_{18}$	$T_{3}$	$T_{9}$
Gini-based importance	0.233	0.198	0.153	0.149	0.098	0.071	0.032

**Table 2:** Cross-section frames at discharge end feature analysis.

Operating Mode	Average Height	Continuity Degree
Over-burning	0.15	0.62
Normal	0.37	0.70
Under-burning	0.58	0.73

**Table 3:** Example of confusion matrix.

	Mode	Actual			Accuracy	False Alarm Rate	Missing Alarm Rate
		${OM}_{1}$	${OM}_{2}$	${OM}_{3}$			
Early warning	${OM}_{1}$	$M_{11}$	$M_{12}$	$M_{13}$	$\eta_{a}$	$\eta_{f}$	$\eta_{m}$
	${OM}_{2}$	$M_{21}$	$M_{22}$	$M_{23}$			
	${OM}_{3}$	$M_{31}$	$M_{32}$	$M_{33}$			

**Table 4:** Early warning results of operating modes.

**Model**	Mode	${OM}_{1}$	${OM}_{2}$	${OM}_{3}$	$\eta_{a}$	$\eta_{f}$	$\eta_{m}$
Model in [7]	${OM}_{1}$	123	42	15	79.63%	25.71%	17.81%
	${OM}_{2}$	1	130	49			
	${OM}_{3}$	0	3	177			
Model in [13]	${OM}_{1}$	153	14	13	86.85%	9.33%	14.62%
	${OM}_{2}$	2	136	42			
	${OM}_{3}$	0	0	180			
Our model	${OM}_{1}$	162	10	8	94.07%	6.67%	5.56%
	${OM}_{2}$	1	168	11			
	${OM}_{3}$	0	2	178			

## Data Availability

The data used in this study cannot be shared publicly due to confidentiality agreements and industrial privacy restrictions. Data may be available from the corresponding author upon reasonable request and with permission from the data provider.
